# Dynamic tracking of scaphoid, lunate, and capitate carpal bones using four-dimensional MRI

**DOI:** 10.1371/journal.pone.0269336

**Published:** 2022-06-02

**Authors:** Mohammad Zarenia, Volkan Emre Arpinar, Andrew S. Nencka, L. Tugan Muftuler, Kevin M. Koch

**Affiliations:** Department of Radiology, Medical College of Wisconsin, Milwaukee, WI, United States of America; Indiana University School of Medicine, UNITED STATES

## Abstract

A preliminary exploration of technical methodology for dynamic analysis of scaphoid, capitate, and lunate during unconstrained movements is performed in this study. A heavily accelerated and fat-saturated 3D Cartesian MRI acquisition was used to capture temporal frames of the unconstrained moving wrist of 5 healthy subjects. A slab-to-volume point-cloud based registration was then utilized to register the moving volumes to a high-resolution image volume collected at a neutral resting position. Comprehensive *in-silico* error analyses for different acquisition parameter settings were performed to evaluate the performance limits of several dynamic metrics derived from the registration parameters. Computational analysis suggested that sufficient volume coverage for the dynamic acquisitions was reached when collecting 12 slice-encodes at 2.5mm resolution, which yielded a temporal resolution of and 2.6 seconds per volumetric frame. These acquisition parameters resulted in total in-silico errors of 1.9°±1.8° and 3°±4.6° in derived principal rotation angles within ulnar-radial deviation and flexion-extension motion, respectively. Rotation components of the carpal bones in the radius coordinate system were calculated and found to be consistent with earlier 4D-CT studies. Temporal metric profiles derived from ulnar-radial deviation motion demonstrated better performance than those derived from flexion/extension movements. Future work will continue to explore the use of these methods in deriving more complex dynamic metrics and their application to subjects with symptomatic carpal dysfunction.

## Introduction

Connective tissue injuries, structural deformities, and structural integrity loss can be difficult to characterize in single-frame static diagnostic images. As a result, imaging of the moving joint has the potential to provide differentiating diagnostic information for use in orthopedic assessment and longitudinal management of injury and disease [1–9]. Improvements in dynamic image acquisition technology and analytic methods continue to offer new potential mechanisms to harness the diagnostic information contained in dynamic images of moving joints. Dynamic imaging of the wrist is a topic of particular interest in the orthopaedic community. Specifically, it is hypothesized that dynamic analysis of diagnostic images can help uncover the role of extrinsic and intrinsic ligaments in wrist dysfunction [10, 11]. This concept has been previously explored and utilized to qualitatively identify abnormalities in wrist carpal bone movements and their correlation with wrist instability [4, 12, 13].

Though dynamic imaging has been performed using multiple imaging modalities such as x-ray fluoroscopy [3] and 4D CT [12–18], the present study explores dynamic joint profiling using magnetic resonance imaging (MRI). The use of MRI for this purpose has two main advantages; First, MRI is a non-ionizing imaging modality, which simplifies patient risk/reward analyses. Second, compared to CT, MRI is more effective at exposing ligament injuries and subtle differences between types of tissue [19], which might aid in the investigation of wrist instability using 4D imaging. The study seeks to test the aim that dynamic 4D MRI can be utilized to track rotational motion of the scaphoid, lunate, and capitate in a fashion similar to previous demonstrations using 4D CT.

The use of MRI for dynamic analysis of the wrist was previously explored by Foster et al, where MRI wrist scans of healthy individuals were acquired at multiple static positions and the carpal bone displacements were studied through a principal component analysis [20]. Foster et al’s study collected data on an asymptomatic cohort undergoing quasi-static radial–ulnar deviation movements. Using this data, several metrics, including the scaphotrapezium joint and the capitate-to-triquetrum distance were derived from the imaging data and analysed to explore modal trends within the study cohort [21].

In a test cohort of 5 subjects performing unconstrained radial-ulnar and flexion-extension deviations with their dominant hand, the present study demonstrates an approach utilizing a dynamic 4D MRI acquisition of the continuously moving and unconstrained wrist to perform dynamic tracking of individual carpal bones.

Due to the competing trade-offs of spatial resolution/coverage, temporal frame rate, and signal to noise ratio inherent to dynamic MRI, only a sub-volume (slab) of the complete bone volume can be consistently captured and segmented. This necessarily requires the registration of high-resolution static volumes to dynamic sub-volumes.

In the present approach, a slab-to-volume registration was performed using a surface point cloud-based approach. Metrics derived from these dynamic registrations were then used to compute rotation components of carpal bones in a radius-based coordinate system. The accuracy of the resulting dynamic metrics were computationally investigated via in-silico simulation. In addition, metrics derived from volunteer subjects were compared to results of recent 4D-CT dynamic studies.

## Methods

The MRI acquisition in this study deploys an unconventional application of the Liver Acquisition with Volume Acceleration (LAVA Flex, GE Healthcare) pulse sequence to capture high resolution static images and 4D dynamic imaging of the moving wrist.

LAVA Flex is a 3D, Fast Spoiled Gradient Recalled (FSPGR) sequence that collects in-phase and out-of phase echoes for two-point Dixon-based fat-water separation [22]. In the present study, the “Water” image output of this technique is utilized for further analysis. To our knowledge, this is the first application of the 3D LAVA Flex sequence for the study of joint dynamics using MRI.

### MRI acquisition

Imaging data were collected on a GE Heathcare Signa Premier 3T MRI scanner using a 16 channel large flex coil. Five healthy subjects with no prior known wrist pathology or bone disease (2 males and 3 females with an average age of 27±3 years) were recruited into an approved protocol by the Medical College of Wisconsin’s Institutional Review Board (IRB) and provided written consent to participate. Subjects were placed in the MRI bore in a prone “superman” position.

The dominant arm of each subject was placed in the center of the receiver array, using sufficient positioning pads to allow the necessary range of motion for ulnar-radial deviation and flexion-extension motions. A picture of the wrist positioned in the scanner is shown in S1 Fig of the Supporting information. No motion-restriction constraints were utilized. Instead, visual cues were used to pace the motion and the subjects were trained prior to the exam using same visual cues (see the training videos S1 and S2 Videos in the Supporting information).

Static images and 40 dynamic sub-volumes with a temporal resolution of 2.6s were acquired using a 3D LAVA Flex series (The acquisition parameters are summarized in Table 1).

The visual guidance utilized to direct subject motion dictated 3 cycles of motion during the 103 second acquisition duration. This rate of motion was found to cause minimal motion artifacts in the described dynamic 3D acquisition and was a fast enough rate for subjects to complete without discomfort. The de-identified source imaging data used in this study has been made freely available through SimTk.org [https://simtk.org/projects/kinematic_mri]. The current database includes DICOM format of static and dynamic MRI images as well as their DICOM-RTSTRUCT formats of the manually segmented scaphoid, capitate, lunate, and radius from 5 healthy subjects.

**Table 1:** Acquisition parameters of the static and dynamic images.

image	voxel size	acquisition matrix	CS	R	TE	TR	FA	NEX	BW
static	0.9 × 0.9 × 1mm^3^	224 × 224 × 60	1	1	1.7ms	5.3ms	10°	1	417Hz
dynamic	1.6 × 1.6 × 2.5mm^3^	128 × 128 × 12	1.4	2	1.2ms	4ms	10°	1	977Hz

Compressed Sensing (CS), Acceleration Factor (R), Time to Echo (TE), Repetition Time (TR), Fractional Anisotropy (FA), Number of Excitation (NEX), Bandwidth/Pixel (BW).

**Fig 1:**
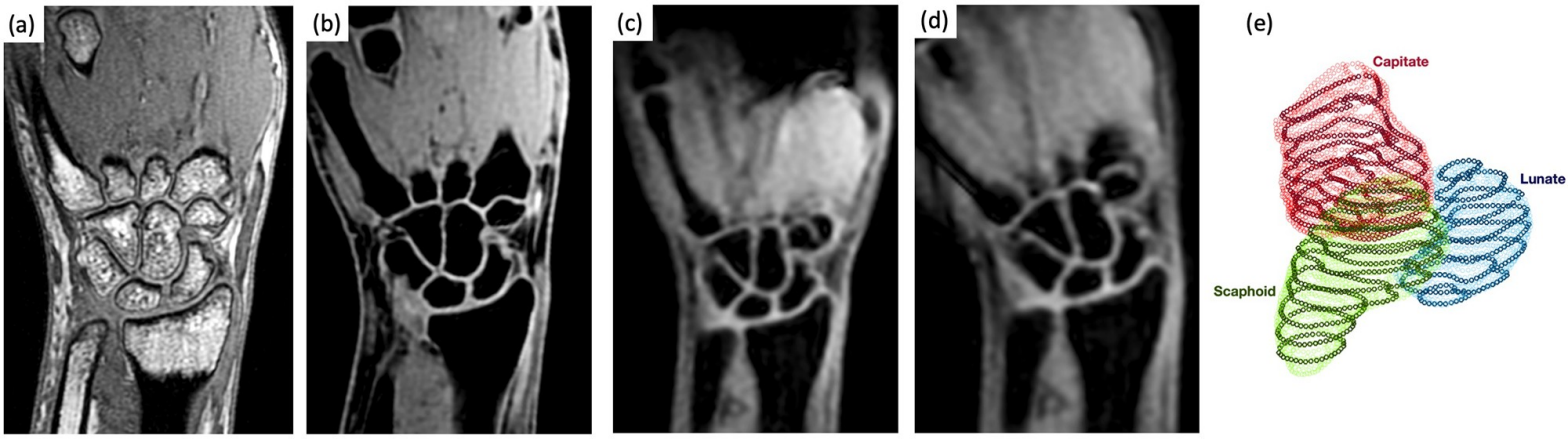
Sample static and dynamic MR images and registered point clouds. Orthogonal imaging planes through the carpal bones of (a) 3D SPGR and (b) LAVA Flex acquisitions. LAVA Flex water-decomposed images were used manually segment the bones of interest. (c,d) Sample slices of 3D dynamic LAVA Flex images utilized to track the static segmentation. (e) Resulting point cloud of boundary points derived from the high-resolution segmentation. The thick points show the boundaries of the moving slices which are registered to the static point clouds.

Fig 1(a)–(d) provide demonstrative images from one subject. High resolution static images are shown in Fig 1(a) and 1(b) obtained using 3D SPGR and LAVA Flex MRI acquisitions, respectively. The LAVA Flex water (i.e. fat-suppressed) reconstructed image series provided excellent bone/tissue contrast, which is essential for accurately segmenting the carpal bones. Two sample images from the 3D dynamic LAVA Flex series during an ulnar-radial deviation are shown in Fig 1(c) and 1(d).

When utilizing dynamic MR acquisition parameters that provided sufficient temporal resolution with minimal motion artifacts, the average number of encoded slices (slab thickness) during the dynamic imaging sequence was a limiting performance factor. For ulnar-radial deviation, average slab coverage was respectively 88±7.8, 95±7.7, and 90±7.2 percent of the scaphoid, capitate, and lunate bones. During the flexion-extension motion, lower bone coverage (slab thickness/bone length) was acquired, with an average values of 70.1±9.8 percent of scaphoid, 98±12.9 percent of capitate, and 83±9.7 percent of lunate.

### Registration approach

Mapping sub-volumes to a given volumetric (3D) reference image is a challenging image registration problem [23]. Here, this problem is addressed using a point-cloud based registration algorithm.

Manual segmentation of the static and dynamic scaphoid, lunate, capitate bones was performed using OHIF viewer [24]. Registration of dynamic to static point clouds derived from the OHIF RTStruct DICOM segmentation outputs was performed in MATLAB [25] using its iterative-closest-point registration function, *pcregistericp*. This function accepts the moving and static point clouds as inputs and performs a 6-degrees of freedom rigid-body transformation by minimizing the root-square-mean-square error (RMSE) of the Euclidean distance between the coordinates of the matched pairs defined by,

$$\text{RMSE}={\left(\frac{\sum_{i=1}^{N_{m}}{\left|R_{m}-R_{f}\right|}^{2}}{N_{m}}\right)}^{1/2},$$ (1)

where, **R**_*m*_ and **R**_*f*_ are the coordinates of the nearest pairs in the moving and static point clouds, respectively. *N*_*m*_ is number of boundary points of the moving point cloud.

The outputs of the registration function are a 4 × 4 transformation matrix, the registered surface point cloud of the moving slab that has been registered to the surface point cloud of the static volume (Fig 1e), and the resulting RMSE. The transformation matrix consists of a 3 × 3 rotation sub-matrix and 3 spatial transformation components.

**Fig 2:**
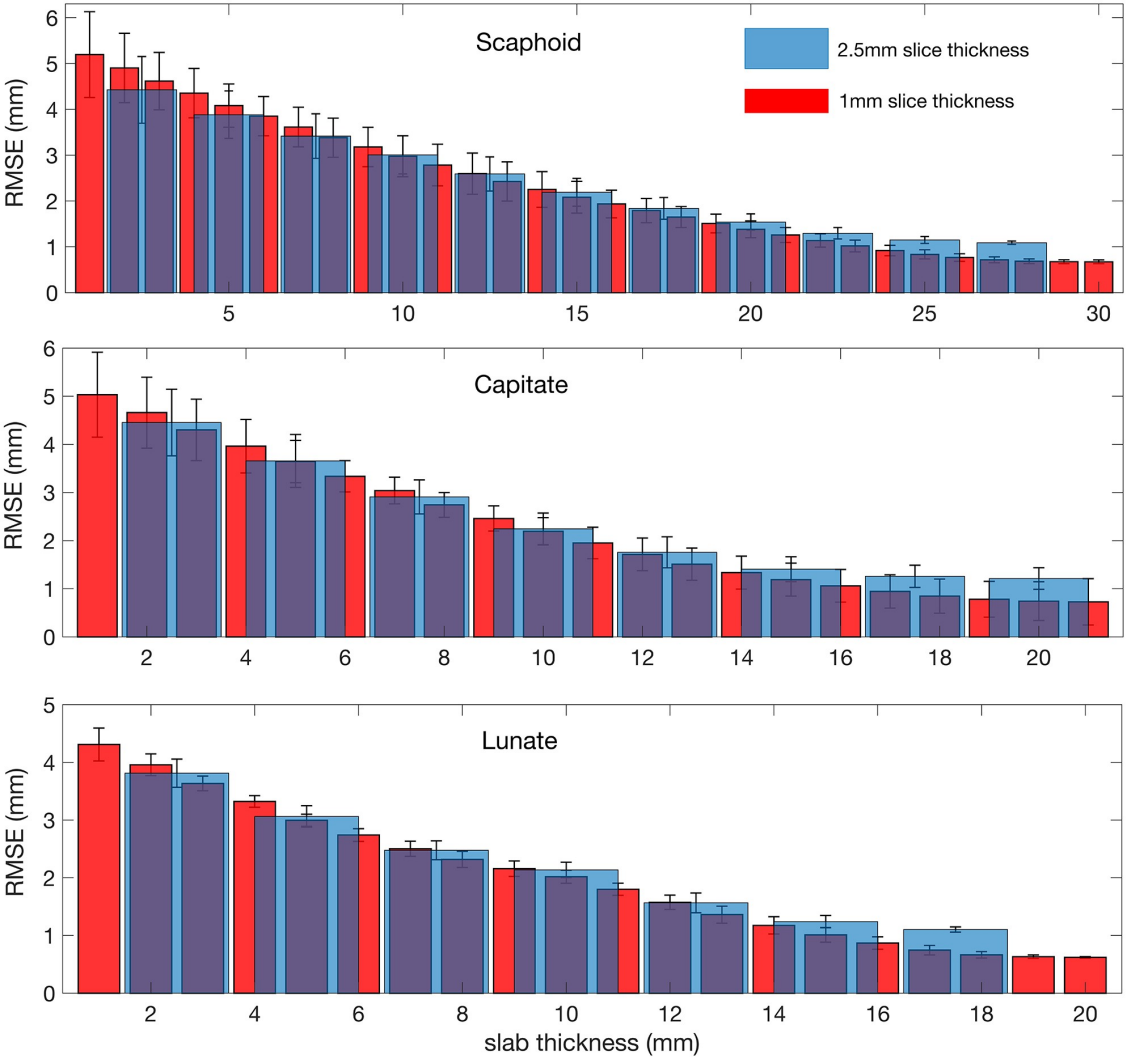
Root-Mean-Square-Error (RMSE) of registration. RMSE of the point cloud registration, Eq (1), averaged over 10 different time frames and for all possible slab locations as functions of slab thickness for 1mm and 2.5mm slice thicknesses of moving slabs of scaphoid, capitate, and lunate. Error bars indicate the standard deviation.

The performance of this registration is related to the slice thickness and number of collected slices (i.e. the acquired slab thickness) for each bone. As expected, when the the slab thickness is increased, the registration performance improves (Fig 2). These findings also demonstrate that slab thickness (bone coverage) has a greater impact on registration performance than slice resolution. The RMSE difference between slabs with 1mm and 2.5 slice thicknesses is found to be less than 0.5mm (i.e. less than the 1mm slice thickness of the static volume) at slab thicknesses with more than 70% bone coverage—which is the case for the data collected and analyzed in this pilot study.

### In-silico error analysis

**Fig 3:**
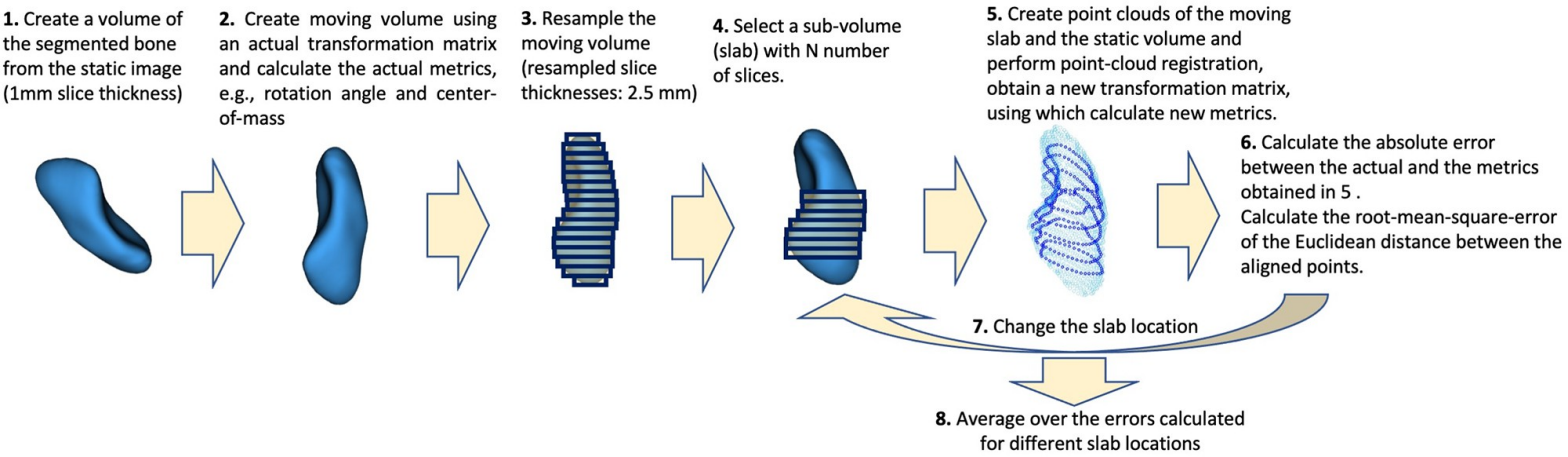
Workflow for processing of an in-silico error analysis in each time frame.

The error analysis process used to calculate the registration error and the resulting in-silico errors in reported metrics is depicted in a flowchart in Fig 3. The high-resolution static volume is first transferred to the moving reference frame using the inverse of the transfer matrix obtained through the registration of the segmented moving slab to the static volume. The resulting moving volume is then resampled to create a moving volume with 2.5mm slice thicknesses (i.e. the same slice thickness as the actual moving slabs). From the resampled moving volume, a sub-volume with *N* slices is chosen (*N* is the number of segmented slices in actual moving images). The value of *N* and location of sub-volume with respect to the full bone was varied for this analysis. Using this synthetically generated sub-volume, a new registration matrix was calculated with respect to the original high-resolution image. The in-silico errors were calculated between the metrics derived from the actual motion and this synthetic data, such as rotation angles, as well as the RMSE between the registered and moving slab. Of note, the presented error analysis provided an in-silico error estimate of the performance of registration approach, whereas an in-vivo true accuracy of the derived dynamic metrics may require experiments on cadaveric samples [26].

## Motion analysis

**Fig 4:**
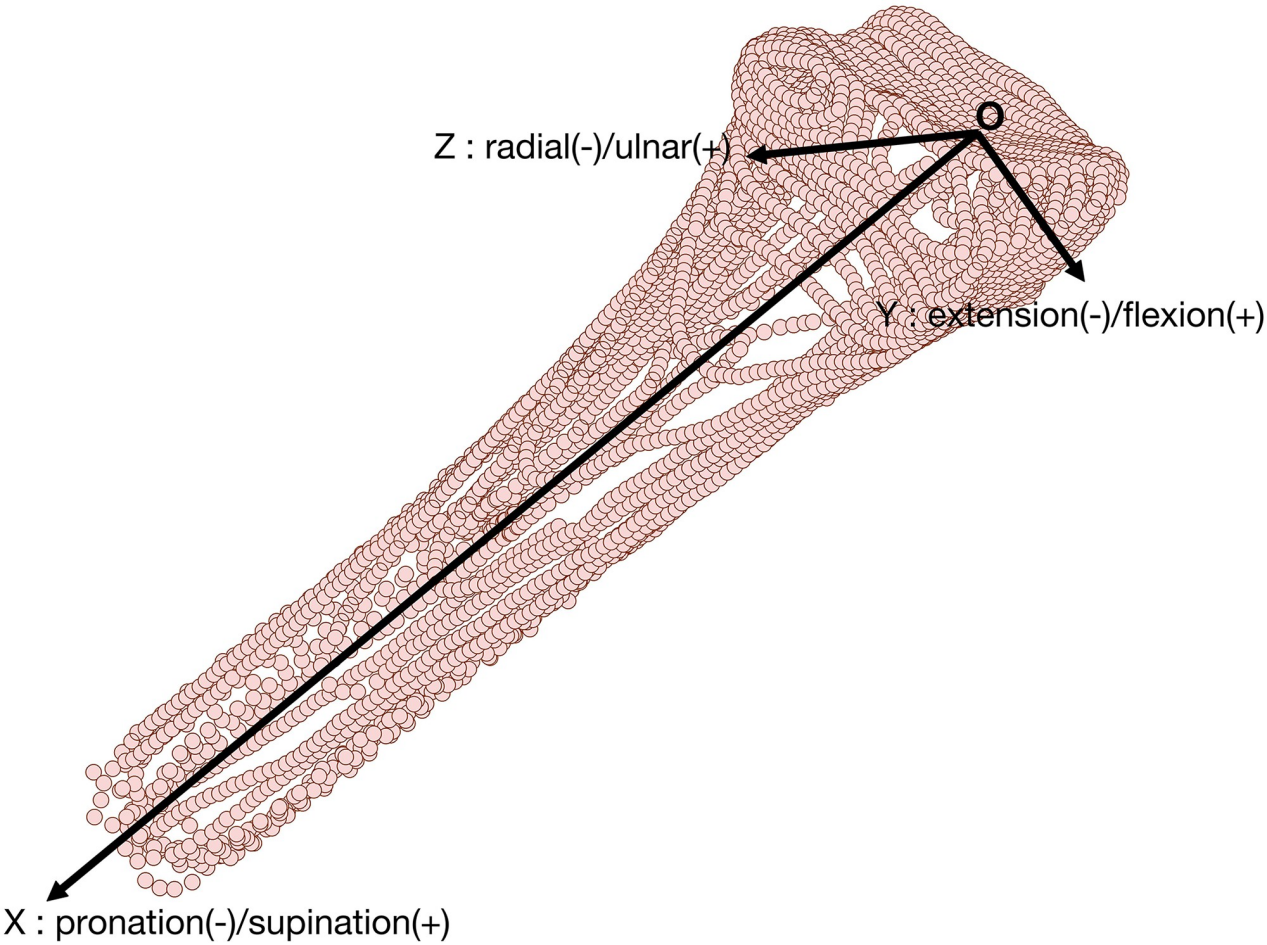
Radial coordinate system. An anatomically defined coordinate system was constructed on the registered point cloud of radius at each cycle position.

To describe the rotational parameters of the scaphoid, lunate, and capitate bones, an anatomically-based coordinate system was referenced to the distal radius using anatomic features of the radial surface (Fig 4). This radius coordinate system (RCS) is a modification of the International Society of Biomechanics (ISB) recommendation [27] and has been used in several recent dynamic studies of wrist carpal bones [18, 28–30].

In the present study, a subject-specific RCS was constructed using the following procedure as described in Ref. [30]. First, a linear best-fit of the centroids of the radial diaphysial cross-sectional contours was used to define the *X*-axis (−*X* indicates pronation and + *X* indicates supination). The intersection of the radial styloid peak orthagonal to the *X*-axis then was used to define the *Y*−axis (−*Y* is extension and + *Y* is flexion) and the cross-product of *X*-axis and *Y*-axis was used to define the *Z*-axis (−*Z* is radial and + *Z* is ulnar deviation). The origin of the RCS was defined as the intersection of the *X*-axis and the distal radial surface. Due to the unconstrained nature of motion in this study and potential motion of the radius contaminating the RCS frame, the static radius volume was also registered to the dynamic sub-volumes of radius. An unique RCS was then constructed using the radial position at each time frame. To describe the rotational parameters of carpal bones in RCS, a helical axis of rotation was first calculated using an angle-axis representation of the rotation matrix obtained through the registration output at each time frame. Angle-axis representation characterizes the rotational parameters of a rigid-body as a single rotation angle *θ* about a unique helical axis [31]. The total rotation angle *θ* can also be represented in terms of a quaternion (see Eq. (18) in Ref. [31]).

Next, a screw axis was defined as *θ****n*** where *n* is the direction of the helical axis. The rotational parameters in radial/ulnar (Z), flexion/extension (Y), and pronation/supination (X) were estimated based on components of the screw axis in the RCS using *θ****n*** · ***n***_*X*_, *θ****n*** · ***n***_*Y*_, and *θ****n*** · ***n***_*Z*_, where ***n***_*X*,*Y*,*Z*_ are the unit vectors along the RCS axes. Components of the screw axes were defined as being parallel to RCS when the wrist is in a neutral position (rotation components are zero in neutral position). Wrist motion was defined as the capitate motion relative to the radius which has been validated in previous studies showing that the capitate and third metacarpal bone rotated together during the radial/ulnar and flexion/extension motions [30, 32]. Using capitate rotation data relative to the radius, the mean wrist motion velocity (wrist movement rate) across the 5 subjects was estimated as 0.94±0.59 degree/second during the radial-ulnar deviation and 0.97±0.74 degree/second during the flexion-extension motion. The mean motion velocity for each subject is shown in Supporting information (see S2 Fig).

## Results

**Fig 5:**
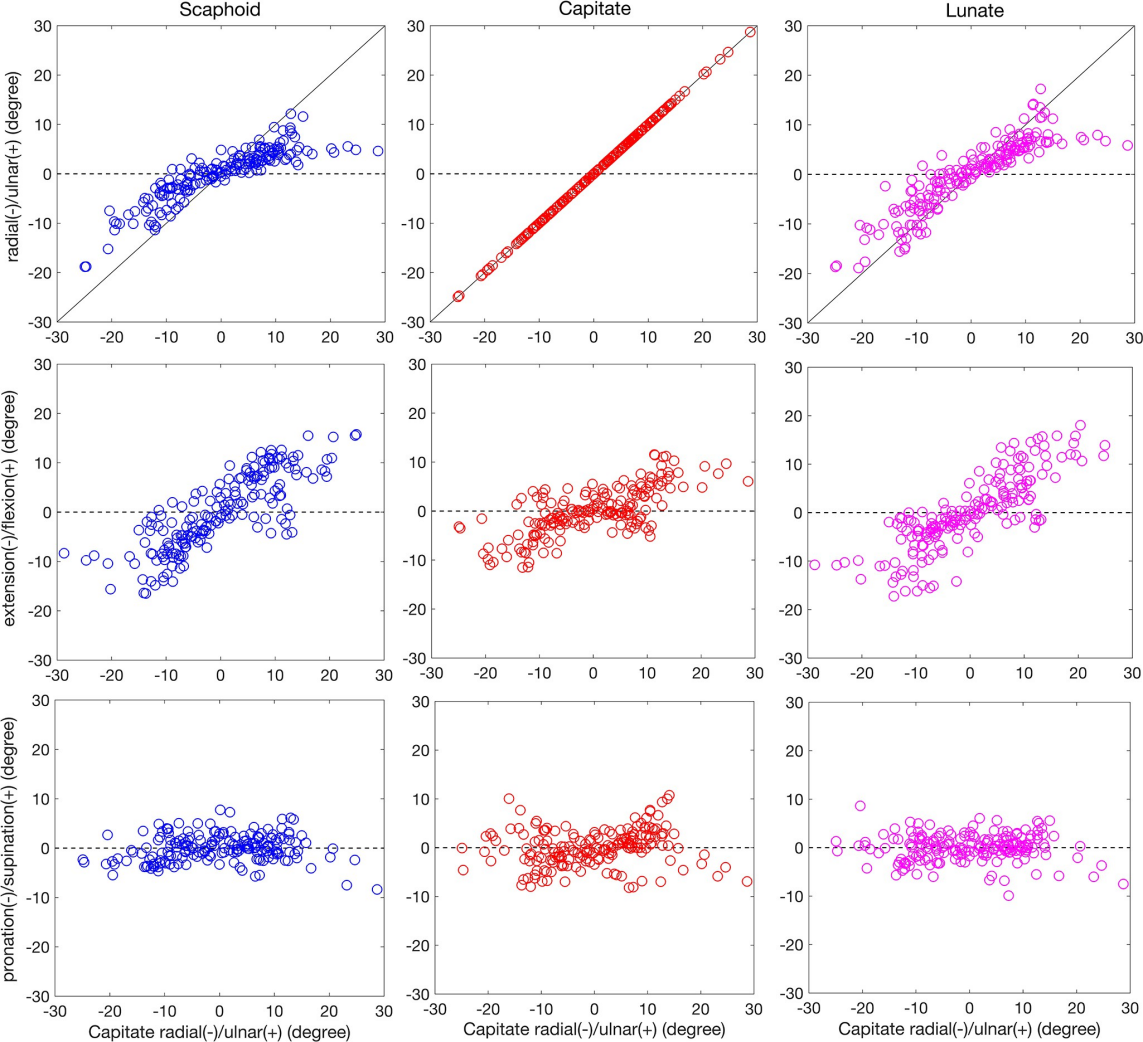
Carpal bone rotational components relative to RCS during radial/ulnar deviation. Scaphoid, capitate, and lunate average flexion/extension, radial/ulnar, and pronation/supination components of rotation for 5 subjects were calculated as a function of the wrist global motion during the radial/ulnar deviation. The global wrist motion was defined by the capitate rotations around the radial/ulnar axis. As a reference, the black line in upper plots represents capitate motion with a slope of 1.

**Fig 6:**
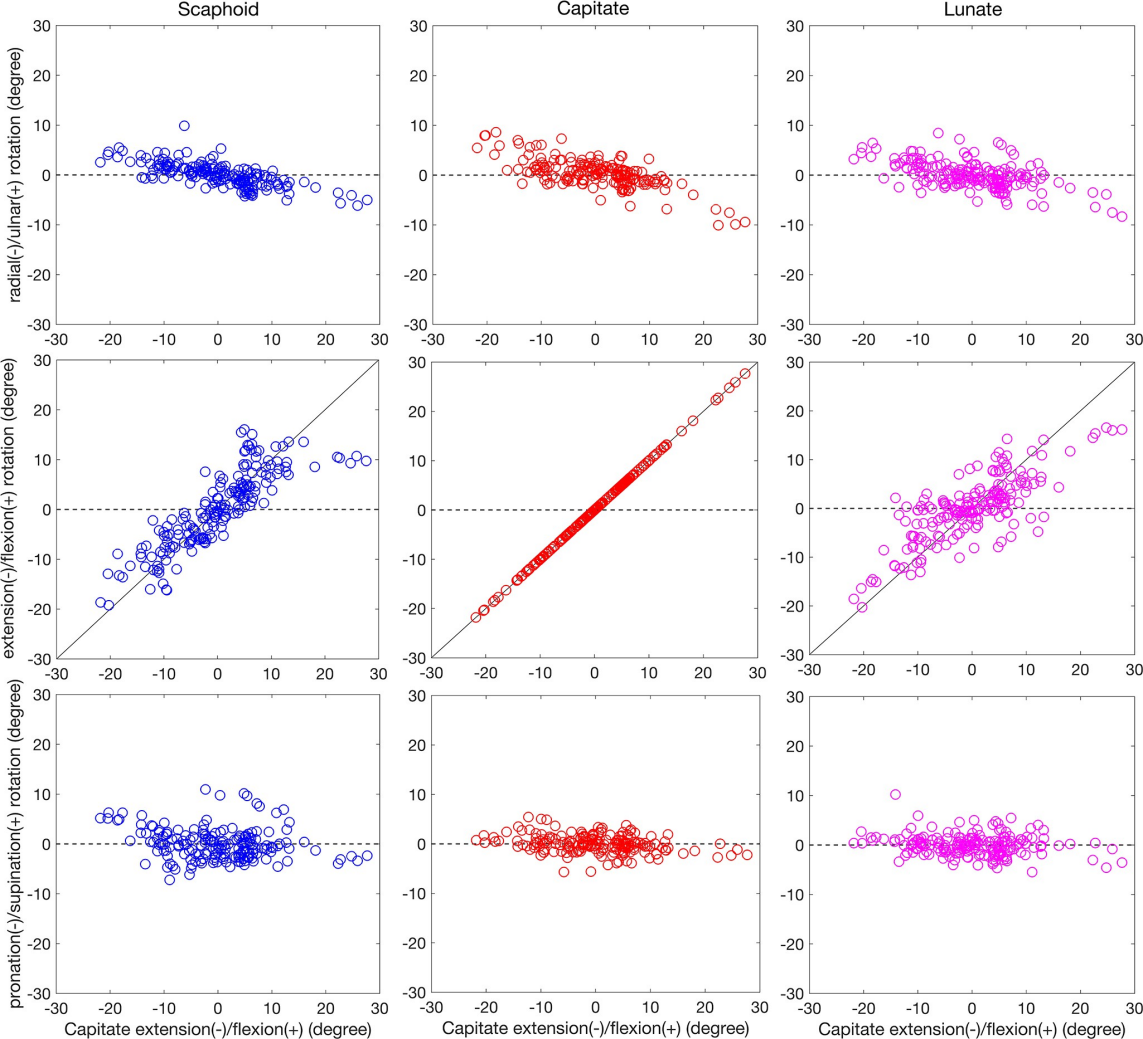
Carpal bone rotational components relative to RCS during flexion/extension motion. Scaphoid, capitate, and lunate average flexion/extension, radial/ulnar, and pronation/supination components of rotation for 5 subjects were calculated as a function of the wrist global motion during the flexion-extension motion. The global wrist motion was defined by the capitate rotations around the extension/flexion axis. As a reference, the black line in middle plots represents capitate motion with a slope of 1.

Carpal bone rotations about the RCS axes were plotted as a function of capitate rotation during the radial-ulnar (Fig 5) and flexion-extension (Fig 6) motions. As visualized in these plots, during the ulnar-radial deviation of the wrist, scaphoid, capitate, and lunate extended when the wrist is ulnar deviated and flexed when the wrist is radial deviated (Fig 5). Carpal bones underwent ulnar deviated during flexion of wrist and radial deviation under wrist extension movements (Fig 6).

During both motions and for all the three carpal bones, rotation about the pronation/supination axis was less significant than the rotations about the ulnar/radial and flexion/extension axes.

Table 2 provides in-silico error analysis results of the principal angle of rotation *θ* around the helical axis of each bone as functions of temporal coverage and number of acquired moving slices in each time frame. Dynamic profiles of the principal rotation angles of scaphoid, capitate, and lunate were provided in S3 Fig of the Supporting information.

**Table 2:** In-silico errors of principal angle of rotation around the helical axis for different acquired slices and temporal resolutions.

acquired slices	temporal resolution (s)	rotation angle error (degree)					
		ulnar-radial			flexion-extension		
		S	C	L	S	C	L
4	2.1	30.5±15.6	17.7±12.3	50±15.6	42.8±19.2	19.5±16.5	56.5±19.2
8	2.3	6.3±3.8	1.8±1	10.1±8	21.6±14.9	4.1±2.4	16.1±15.4
**12**	**2.6**	**3.6±2.7**	**0.6±0.4**	**2.7±1.9**	**7.2± 5.2**	**2.2±+1.9**	**2.4±1.7**
16	3.2	2.3±1.9	0.5±0.4	2.1±1	3.1±2.6	2.1±1.9	1.7±0.9
20	3.7	2.2±1.9	0.6±0.4	2.1±1	2.7±2.2	2.1±1.9	1.7±0.9

Due to slice overlapping (to prevent foldover aliasing artifacts), 4 of the initially acquired slices in each volume were removed (2 on each side of the encoded slab). The preliminary dynamic profiling of carpal bones in this study was therefore performed using 12 slices (out of 16 initially acquired slices). This case is highlighted in bold text. The error in each case is obtained by averaging errors computed over the 200 time frames of 5 subjects.

The reduced bone volume coverage for the scaphoid (70.1%±9.8%) compared to the capitate (98%±12.9%) and lunate (83%±9.7%) during the flexion-extension motion is the most probably cause of the higher in-silico errors (7.2°±5.2°) for the scaphoid.

Using reduced encoded slices to enable temporal resolutions greater than 2.6s/frame, resulted in significantly increased errors. As expected, for lower temporal resolutions using higher numbers of encoded slices per frame, the registration accuracy improved. However, empirical testing uncovered difficulty for subjects to move their wrists at such a slow rate. Therefore, the utilized approach (12 2.5mm slices acquired at 2.6s/frame) provided an optimal balance of subject comfort, image/data quality, and registration accuracy.

## Discussion

Using advanced 4D MRI and point-cloud registration techniques, the motions of scaphoid, capitate, and lunate carpal bones within 5 healthy subjects were analyzed during ulnar-radial deviation and flexion-extension movements. In silico error analyses were performed for different MRI acquisition parameter settings, with computational results suggesting that acceptable volume coverage for the dynamic acquisitions was achieved when collecting 12 slice-encodes at 2.5mm resolution, yielding a temporal resolution of and 2.6 seconds per volumetric frame. These acquisition parameters resulted in in-silico errors of 1.9°±1.8° in derived principal rotation angles dynamic profiles within ulnar-radial and 3°±4.6° within flexion-extension motion and our results for the rotation components of carpal bones in radius coordinate system are reliable in this range.

The preliminary dynamic profiles derived within this exploratory study are encouraging, Profiles derived from this study agree with the recent similar studies using dynamic 4D-CT imaging of healthy subjects. In particular, the present findings agree with those of Brinkhorst et al, [18], Foumani et al, [33] and of Crisco et al., [30]. In addition, the present results suggested that scaphoid and lunate rotate linearly with the wrist rotation during the flexion-extension motion, which agrees with results derived from recent cadaveric studies [34].

Although 4D CT imaging provides better temporal and spatial resolution than MRI, the non-ionizing radiation and ability of MRI to capture high-contrast images of soft tissues and connective structures makes it an appealing option for for the dynamic analysis of moving joints.

It is also important to note that the unique MRI acquisition approach demonstrated in this study does not use any prototype pulse-sequences and is commercially available on most GE Healthcare scanner platforms. Similar sequences and capabilities are also available on other vendor platforms.

There are several limitations to the present study; *i*) Because of the narrower field of view in the sagittal plane, less bone coverage and thus higher in-silico errors were obtained for the flexion-extension motion. Continued technology development of the deployed 4D MRI methodology will be required to surpass the dynamic image resolution. *ii*) The sample of this preliminary feasibility study was small and was only intended for demonstration purposes. Larger cohort studies will be required for more advanced analysis of specific dynamic profile behavior and trends. *iii*) Previous studies have been shown that carpal bones exhibit hysteresis that is dependent on the direction of wrist motion [35]. The present analysis did not account for this effect, which was shown to be less significant in normal wrists [35]. *iv*) We did not investigate a possible relationship between carpal bone morphology and joint dynamics, which would require a larger study cohort. *v*) Despite the fact that no external device was used to control the wrist motions, reaching the extreme radial/ulnar and flexion/extension positions may have been limited by the MRI radiofrequency reception coil that was used to capture the static and dynamic images. As a result, a lower range of wrist motion was observed when compared to previous dynamic studies using CT [29, 30] or external motion capture systems in cadaveric samples [34, 36].

In addition to study of carpal bones rotations, many more metrics such as gaps between the carpal bones and their center-of-mass displacements could be tracked in similar fashion. Future work will continue to explore the use of the new methods presented in this preliminary study.

## Supporting information

S1 Fig Unconstrained wrist positioning in the flex coil during scanning of static and moving images. (TIFF)

S2 Fig Wrist motion velocity (degree of movement/second) averaged over 40 dynamic frames in each subject. The error bars indicate the standard deviations. The velocities were obtained using the capitate rotation relative to the radius.
(TIFF)

S3 Fig Dynamic profiles of scaphoid, capitate, and lunate total rotation angles about the helical axis in relation to their starting positions. The in-silico errors are represented by the shaded error bands, calculated using the process shown in Fig 3. To obtain the principal rotation angles *θ*, see Sec. Motion analysis in the main text, with respect to the initial position, the high-resolution static volume was first registered to the initial moving slab and the resulting volume was then utilized to register the other moving slabs. In order to clarify the modal nature of the motion, a smoothing filter is applied. The smoothing was performed in MATLAB using *rloess* method with a span value of 20% of the total number of points. This method assigns zero weight to data outside six mean absolute deviations.
(TIFF)

S1 Video Training video for ulnar-radial motion. The subjects were trained to pace the motion of this video during capture of dynamic images of ulnar-radial motion.
(MOV)

S2 Video Training video for flexion-extension motion. The subjects were trained to pace the motion of this video during capture of dynamic images of flexion-extension motion.
(MOV)
