# Supplementary figures and images for: Dynamic tracking of scaphoid, lunate, and capitate carpal bones using four-dimensional MRI
Source: PLoS One. 2022 Jun 2;17(6):e0269336. doi: 10.1371/journal.pone.0269336 (PMC9162359; doi:10.1371/journal.pone.0269336)

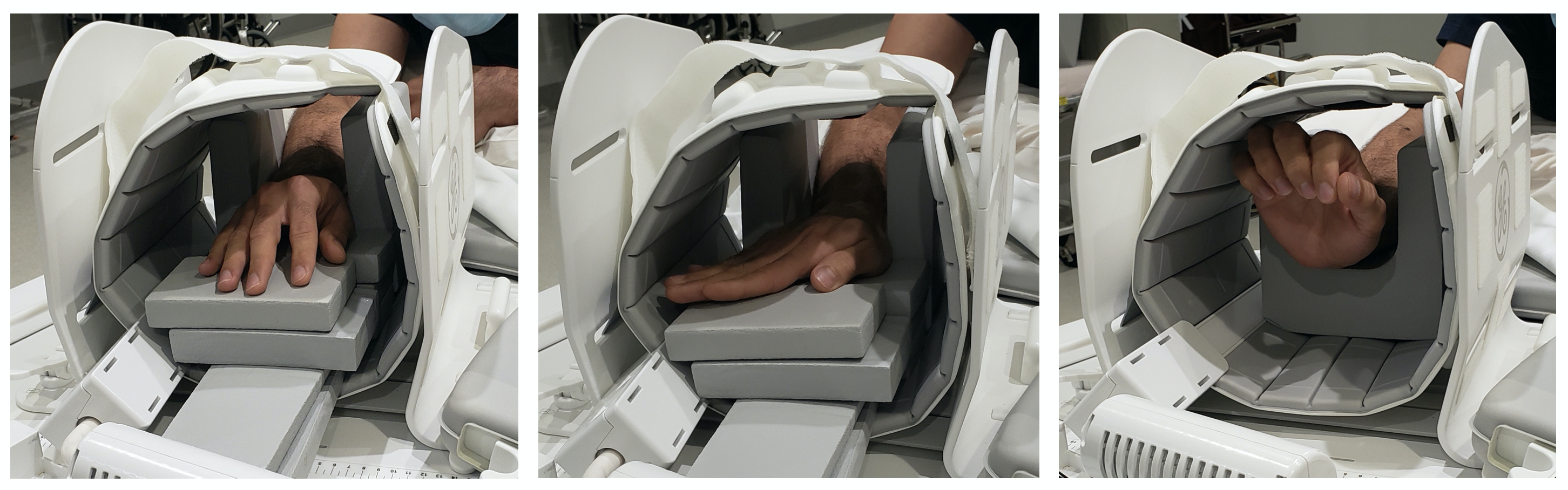

Supplement: S1 Fig — (TIFF) [file pone.0269336.s001.tiff]
